# An exploration of mechanism of high quality and yield of *Gastrodia elata* Bl. *f. glauca* by the isolation, identification, and evaluation of *Mycena*

**DOI:** 10.3389/fmicb.2023.1220670

**Published:** 2023-10-19

**Authors:** En Yu, Qun Liu, Yugang Gao, Yaqi Li, Pu Zang, Yan Zhao, Zhongmei He

**Affiliations:** ^1^College of Chinese Medicinal Materials, Jilin Agricultural University, Changchun, China; ^2^Institute of Botany, Jiangsu Province and Chinese Academy of Sciences (Nanjing Botanical Garden Memorial Sun Yat-Sen), Nanjing, China

**Keywords:** *Gastrodia elata* Bl. *f. glauca*, *Mycena*, separation and identification, nutrition elements, microbial diversity

## Abstract

*Gastrodia elata* Bl. *f. glauca* is an important traditional Chinese medicinal plant. The yield and quality of *Gastrodia elata* Bl. have significantly decreased due to multigenerational asexual reproduction. Therefore, it is necessary to have sexual reproduction of *Gastrodia elata* Bl. to supplement the market supply. Seeds of *G. elata* Bl. have no endosperm, and their sexual reproduction depends on the nutrients provided by the embryo cells infected by *Mycena* fungi to complete seed germination. However, *Mycena* fungi are small and have many species, and not all *Mycena* fungi can promote the germination of *G. elata* Bl. seeds. Therefore, it is of great significance to isolate and identify suitable germination fungi and explore the mechanism for improving the production performance and yield, and quality of *G. elata* Bl. Six closely related *Mycena* isolates, JFGL-01, JFGL-02, JFGL-03, JFGL-04, JFGL-05, and JFGL-06, were isolated from the leaves and protocorms of *G. elata* Bl. *f. glauca* and were identified as *Mycena purpureofusca*. The mycelial state and number of germinating protocorms were used as indicators to preferentially select *Mycena* fungi, and it was concluded that JFGL-06 had the best mycelial state and ability to germinate *G. elata* Bl. seeds. Finally, a mechanism to increase the yield of *G. elata* Bl. was explored by comparing the changes in nutrient elements and microbial diversity in the soil around *G. elata* Bl. with different strains. JFGL-06 proved to be an excellent *Mycena* fungal strain suitable for *G. elata* Bl. *f. glauca*. Compared with the commercial strain, JFGL-06 significantly increased the C, N, Na, Mg, S, Cl, K, Ca, and Fe contents of the soil surrounding the protocorms of *G. elata* Bl. *f. glauca*. JFGL-06 improved the composition, diversity, and metabolic function of the surrounding soil microbial community of *G. elata* Bl. *f. glauca* protocorms at the phylum, class, and genus levels, significantly increased the relative abundance of bacteria such as *Acidobacteria* and fungi such as *Trichoderma* among the dominant groups, and increased the abundance of functional genes in metabolic pathways such as nucleotide metabolism and energy metabolism. There was a significant reduction in the relative abundance of bacteria, such as *Actinomycetes,* and fungi, such as *Fusarium,* in the dominant flora, and a reduced abundance of functional genes, such as amino acid metabolism and xenobiotic biodegradation and metabolism. This is the main reason why the JFGL-06 strain promoted high-quality and high-yield *G. elata* Bl. *f. glauca* in Changbai Mountain.

## 1. Introduction

*Gastrodia elata* Bl. *f. glauca*, known as Tianma in Chinese, is a perennial monocotyledonous plant mainly found in the Changbai Mountain region of Jilin Province, China. Its dried tubers are often used as valuable traditional Chinese medicines with various modern pharmacological properties, such as neuromodulation (Lin et al., 2018; Liu Y. et al., 2018), treatment of depression, Alzheimer’s disease (Doo et al., 2014; Huang et al., 2016; Ng et al., 2016; Liu B. et al., 2018; Ding et al., 2019), and memory improvement (Chen et al., 2011; Ding et al., 2019). *G. elata* Bl. *f. glauca* is an excellent variety of *G. elata* Bl. and its demand has risen sharply in recent years. However, the wild *G. elata* Bl. resources are rapidly becoming scarce and need to be planted artificially to meet the demand. Multiple generations of asexual reproduction in *G. elata* Bl. have resulted in a significant decrease in both yield and quality, leading to a loss of cultivation value. Therefore, sexual reproduction of *G. elata* Bl. is particularly important.

*Mycena* fungi are essential symbionts for the production of *G. elata* Bl. and belong to the Basidiomycota family. *Mycena*, mainly includes the three genera *Mycena osmundicola*, *M. dendrobii,* and *M. orchidicola* (Kim et al., 2006; Zeng et al., 2017, 2018). Seeds of *G. elata* Bl. have no endosperm, and their sexual reproduction depends on the nutrients provided by the embryo cells infected by *Mycena* fungi to complete seed germination (Zeng et al., 2017; Li et al., 2020). Therefore, it is necessary to isolate additional strains of *Mycena*. However, there are many difficulties in the process of isolating *Mycena* fungi, such as their small morphology and many species, as not all *Mycena* fungi can promote the germination of *G. elata* Bl. seeds. At the same time, the survival time of the fruiting body is inconsistent with the growth period of *G. elata* Bl., which makes it particularly difficult to isolate germinating fungi. Therefore, it is difficult to isolate and identify *Mycena* fungi suitable for the germination of *G. elata* Bl. *f. glauca* seeds.

The symbiotic relationship between orchid plants and mycorrhizal fungi enables the plants to acquire a significant amount of microbial elements, including N, P, K, and other nutrients, from their surroundings. In particular, the presence of arbuscular mycorrhizal fungi (AMF) has been found to enhance the growth and nutrient absorption capabilities of plants, such as N, P, and other essential nutrients (Barazetti et al., 2019). Previous studies have demonstrated that when soybean and other leguminous plants are inoculated with arbuscular mycorrhizal fungi (AMF) in soils contaminated with heavy metals, it can lead to inhibition of metal absorption, reduction of their toxicity, and enhancement of metal tolerance (Antunes et al., 2006; Babajide et al., 2008; Suri and Choudhary, 2013). Symbiotic strains of plants that exhibit excellent interactions often have distinct roles compared to other strains. These distinct roles enable them to facilitate the absorption of nutrients by the host plants. Therefore, to understand the reasons behind the excellence of these strains, it is important to investigate the changes in the elemental content of plants and the surrounding soils.

The soil microbial diversity is a valuable supplement that affects plant growth and development (Khourchi et al., 2022). Symbiotic strains can affect the structure and function of soil microbial communities in the plant rhizosphere, thus indirectly affecting the nutrient absorption and disease resistance of host plants (Khourchi et al., 2022). Some studies have shown that some bacteria can promote the growth of *G. elata* Bl. and *Armillaria* in the rhizospheres of *G. elata* Bl. (Liu et al., 2022) and some non-mycorrhizal fungi in the roots of orchids can antagonize pathogenic bacteria in the soil (Ma et al., 2015; Waud et al., 2016). However, it is necessary to further explore whether *Mycena* fungi have specific effects on soil microorganisms around the protocorm of *G. elata* Bl. and whether they promote the growth of beneficial microorganisms and inhibit the survival of harmful microorganisms in *G. elata* Bl.

The objectives of this study were to isolate and identify several *Mycena* strains, screen for an excellent *Mycena* strain using different evaluation indicators, and explore the effects of different *Mycena* strains on nutrient absorption in the *G. elata* Bl. protocorm and its surrounding soil. Finally, we explored the effects of different germination strains on the microbial diversity of *G. elata* Bl. protocorm surrounding the soil. Therefore, we have found some of the important factors that may be associated with high quality and high yield of *G. elata* Bl.

## 2. Materials and methods

### 2.1. Isolation of *Mycena* fungi

In this study, we first isolated and identified *Mycena* fungi from the leaves of wild *G. elata* Bl. *f. glauca* growing sites and wild *G. elata* Bl. *f. glauca* protocorms collected from the Changbai Mountain region of Jingyu and Huinan counties in Jilin Province, China in 2019. The collected leaves were first washed with water and soaked in 75% alcohol for 3 min, while the collected protocorms were washed and the surface gently wiped with alcohol wool, both were rinsed 2–3 times with sterile water in an ultra-clean bench, surface water was absorbed from sterile filter paper, cut into small pieces and inoculated onto potato dextrose agar (PDA) medium, followed by incubation at 25°C in a constant temperature incubator. Once the colonies reached a size of approximately 1–2 mm, the white colonies were transferred onto a fresh PDA medium. Any stray colonies were carefully removed and this process was repeated several times until the strain was fully purified. The purified *Mycena* fungi were then inoculated onto the new PDA medium and the growth rate was measured at the same time every day using vernier calipers, while the morphological structure of the mycelium was observed by light microscopy.

### 2.2. Molecular identification of *Mycena* fungi

The full-length region of the fungal ITS was amplified through PCR using the universal primers ITS4F (5’-TCCTCCGCTTATT GATATGC-3′) and ITS5R (5’-GGAAGTAAAAGTCGTAACAAGG-3′) (Toju et al., 2012). The fungal DNA extraction kit produced by Solarbio Technologies Ltd. was used to extract the DNA of the *Mycena* fungi. The PCR reaction system was 12.5 μl 2 × Taq MasterMix for PAGE, 1.0 μl each of upstream and downstream primers (10 μmol/L), 3.0 μl of DNA template, 7.5 μl of deionized water, a total of 25 μl; the PCR warming procedure was: pre-denaturation at 95°C (5 min), denaturation at 95°C (30 s), and annealing at 54°C for 30 s. The PCR procedure was as follows: pre-denaturation at 95°C (5 min), denaturation at 95°C (30 s), annealing at 54°C for 30 s, extension at 72°C for 90 s, 30 cycles, and finally termination at 72°C for 10 min. The amplification products were sent to Bioengineering (Shanghai) Co., Ltd. for sequencing, and the results obtained from sequencing were submitted to the NCBI database for BLAST comparison, phylogenetic analysis was performed using Mega 7.0 software, and the prepared sequences were compared using Clustal-W software (Tamura et al., 2007; Stecher et al., 2020). Finally, to analyze and compare the intraspecific and interspecific genetic distances of the *Mycena* fungi, a phylogenetic tree was constructed using the neighbour-joining method (the bootstrap test of 1,000 times) (Mollaei et al., 2019; Liang et al., 2021).

### 2.3. Field trials

The laboratory isolated and identified various strains of *Mycena* fungi which were later utilized in field experiments with *G. elata* Bl. *f. glauca* seeds provided by Baishan Jingzhen Gastrodia Development Co., Ltd. The same mass of *G. elata* Bl. seeds and the same mass of different strains of *Mycena* fungi were weighed, mixed, and sown in non-woodland fields at the base of Baishan Jingzhen Gastrodia Development Co., Ltd. One strain of *Mycena* fungus was planted in a group with *G. elata* Bl. *f. glauca* seeds, and three parallel groups were set up with 10 replicates in each group. After 2 months, the protocorms were collected from a unit area of 10 × 10 cm^2^, and their weight, number, and length were measured.

### 2.4. Sample collection

For the collection of the soil around the protocorm, first carefully brush the soil on the surface of the protocorm (tightly adhered soil within about 2 mm) with a sterilized soft brush. Next, select the protocorm as the sampling center (2–10 cm) and collect 5–10 g of soil from multiple points surrounding the protocorm. Transfer the collected soil, along with the adhered soil, into a sterile self-sealing bag. Repeat this process three times for each of the different treatments. After the soil is naturally air-dried, impurities such as animal and plant residues, as well as gravels, are removed. The large samples are then mashed, sieved using a 2 mm sieve, and stored for future use.

### 2.5. Degradability test of strains

Under aseptic conditions, single colonies were selected from the isolated and purified parent species. These colonies were then inoculated on PDA plate medium and passed on for six generations, with three replications in each generation. This was done to observe the germination time, growth rate, and colony morphology of the subsequent strains, specifically looking for any signs of decline.

### 2.6. Elemental determination of the protocorms of *G. elata* Bl. *f. glauca* and their surrounding soil

For the subsequent experiments in this study, a high-quality strain of *Mycena* fungi was chosen based on the quality of the cultured protocorms. In the subsequent experiments, this excellent *Mycena* strain was used as the treatment group and the commercial strain of *Mycena* fungi was still used as the control group for the subsequent experiments. The protocorms from both groups were washed and dried to constant weight, crushed, filtered through a 60-mesh sieve, and the soil collected around the perimeter of the protocorms was removed. 0.500 g (accurate to 0.001 g) of each of the protocorms and soil from different groups were accurately weighed in a microwave digestion jar, 5.0 ml of HNO_3_ and 1.0 mL of H_2_O_2_ were added, mixed well, the lid was tightened, and the jar was digested at 120°C according to the standard operating procedure of the microwave digestion apparatus. After the digestion was completed, the jar was cooled down and removed, the lid was slowly opened and the acid was run down to 0.5 ml, then fixed to 50 mL using 1% nitric acid and shaken well. 1% HNO_3_ was used as a blank control solution. Lens voltage: 2.00–25.00 V; extended voltage: −700.00 V; hexapole bias voltage: −1.60 V; quadrupole bias voltage: 0.60 V; atomization gas flow rate: 0.90 L min^−1^; cooling gas flow rate: 1.50 L min^−1^; auxiliary gas flow rate: 0.80 L min^−1^; sample depth: 200.00 mm; repeat three times and take the average value.

### 2.7. Analysis of surrounding soil microbial diversity of the protocorms of *G. elata* Bl. *f. glauca*

According to the results of the field test, the protocorm soil treated with the commercial strain was designated as the control group (a), while the protocorm soil treated with the JFGL-06 strain was designated as the treatment group (b). The soil surrounding the protocorm collected during the field test with different treatments was divided into 2 ml EP tubes, with each tube containing 1–2 g of sample. In total, 6 tubes of samples were collected. The total genomic DNA was extracted from soil samples using the CTAB method. Bacteria were primed with 338F (5′- ACTCCTACGGGAGGCAGCA-3′) and 806R (5′- GGACTACHVGGGTWTCTAAT-3′) as universal primers, and fungi with ITS1F (5’-CTTGGTCATTTAGAGGAAGTAA-3′) and ITS2R (5′- GCTGCGTTCTTCATCGATGC-3′) as universal primers for PCR amplification reactions. Amplification products were collected and purified using an agarose gel DNA purification kit (TaKaRa). Sequencing was performed based on the Illumina HiSeq 2,500 platform. Secondary clustering of each sequence data was then performed using USEARCH (version 10.0) at a quality control level of 97% likelihood (Edgar, 2013), the sequences were divided into OTUs/ASVs by the DADA2 method in QIIME2 (version 2020.6), and Venn diagrams were used to show the amount of data that was co-owned and unique to the sample (Chen and Boutros, 2011). Finally, each column was classified by species type and sequence annotation in the Species Sequence Information Database using the RDP classifier for the bacterial 16S rRNA database Silva (Release 132^1^) and the fungal ITS database Unite (Release 8.0^2^). Species abundance tables at different taxonomic levels were generated using QIIME software and then plotted into community structure maps and species clustering heat maps at each taxonomic level of the samples using R language tools.

### 2.8. Statistical analysis

All statistical analyses of the data were performed by one-way ANOVA, LSD, and Duncan’s test at the *p* < 0.05 level of significance. Sample Alpha diversity indices were assessed using QIIME2 to calculate Ace, Chao1, Shannon, and Simpson indices (Grice et al., 2009), respectively, and Shannon diversity index dilution curves, rank abundance curves, and species accumulation curves were plotted using Mothur software and R language tools (Koljalg et al., 2013). Samples were hierarchically clustered using unweighted pairwise averaging (UPGMA) through the R language tool to produce sample heat maps to determine the similarity of species composition between samples (Lozupone and Knight, 2005). Sample variability between groups was analyzed by ANOVA (analysis of variance) and LEfSe (analysis of significant species differences between groups) (Segata et al., 2011). Differences in function between different groupings were analyzed using PICRUSt2 (Parks et al., 2014).

## 3. Results

### 3.1. Isolation and identification of *Mycena* fungi from *G. elata* Bl. *f. glauca*

**Figure 1:**
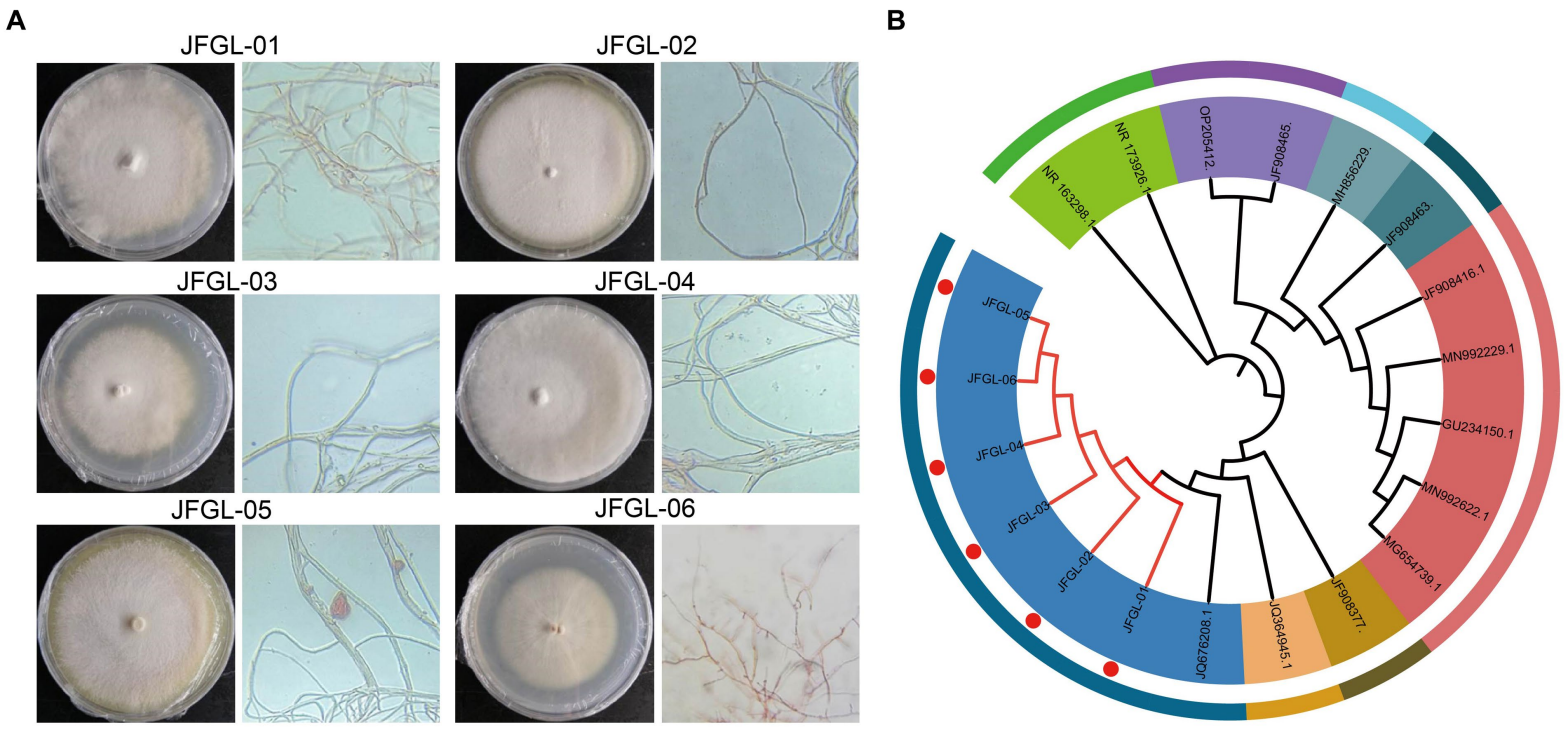
Isolation and identification of germinating fungi. **(A)** Colony morphology and microscopic observation characteristics of six germinating fungi. **(B)** Phylogenetic tree of six germinating fungi. The phylogenetic tree was constructed using the neighbor-joining method (1,000 bootstrap replications). The accession numbers for the sequences retrieved from the GenBank database are listed in Supplementary Table S2.

In this study, we isolated six isolates of *Mycena* fungi from the protocorm and leaves of *G. elata* Bl. *f. glauca*. Specifically, JFGL-01, JFGL-02, and JFGL-03 were found in the leaves while JFGL-04, JFGL-05, and JFGL-06 were identified in the protocorm. Six isolates were observed on a PDA plate medium, with dense mycelial growth. JFGL-06 grew in a counter-clockwise direction, while JFGL-01, JFGL-02, JFGL-03, and JFGL-04 grew in a clockwise direction. JFGL-05 exhibited radial growth. The spore germination time for each strain ranged between 2 and 3 days, and the mycelial growth rate ranged from 0.241 to 0.269 cm/day (Figure 1A and Supplementary Table S1).

A single band ranging in size from 500 to 750 bp was produced through PCR amplification of the ITS rDNA region. The resulting sequences have been registered in the NCBI database (accession numbers MW404447, MW404448, MW404449, MW404450, MW404451, and MW404452, respectively). The BLAST comparison revealed that all six strains were more than 95% identical to the *Mycena purpureofusca* and *Mycena citrinomarginata* (Beye et al., 2018). By further constructing a phylogenetic tree, the results showed that all six strains clustered on the same branch with strains of *Mycena purpureofusca*, indicating that all six strains were *Mycena purpureofusca* (Figure 1B). Six *Mycena* strains will be evaluated subsequently.

### 3.2. Screening for excellent *Mycena* fungi in *G. elata* Bl. *f. glauca*

**Figure 2:**
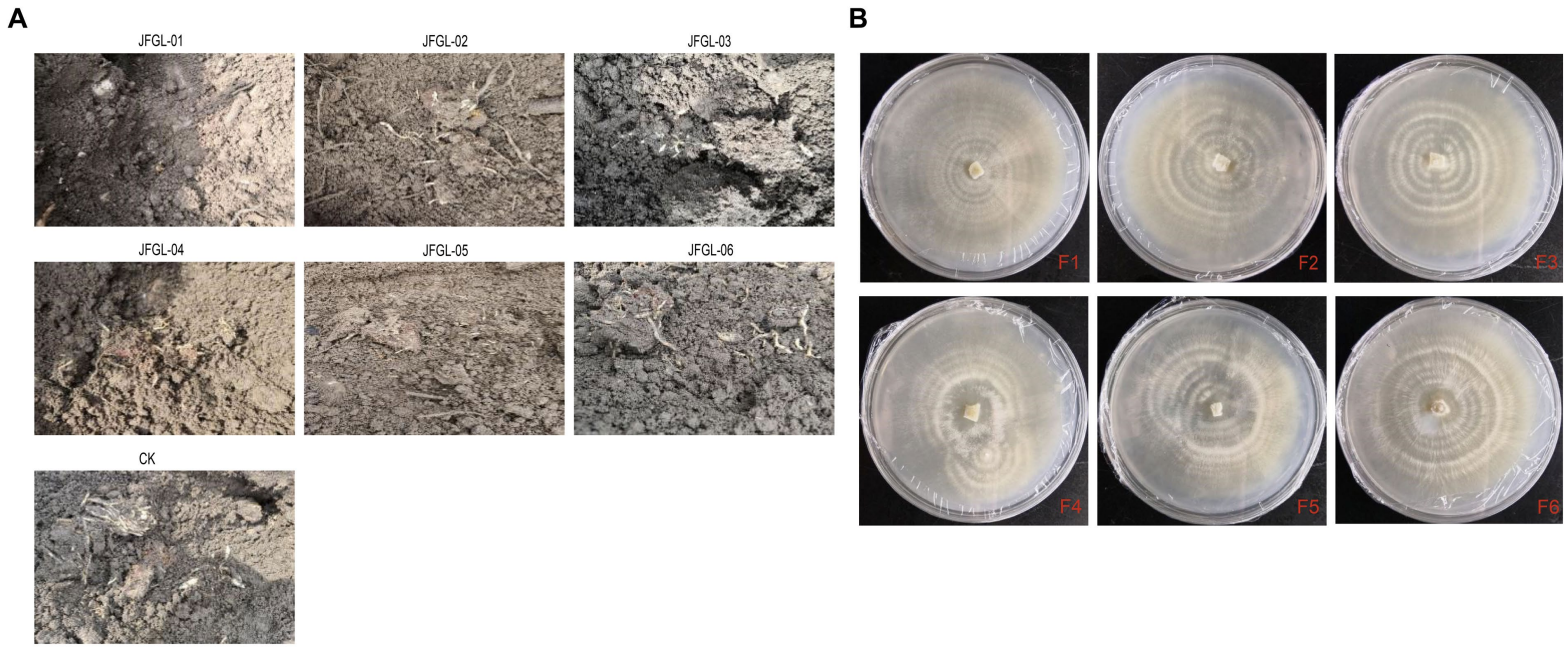
Harvesting realities of protocorms **(A)** and degradation test of strain JFGL-06 **(B)**. CK represents the control commercial strain. F+ numbers indicate the number of passages.

It is crucial to select the most suitable strains of *Mycena* fungi for further research on *G. elata* Bl. seeds, as their germination efficiency varies across different strains. Results from field trials revealed a significant difference (*p* < 0.05) in the number of *G. elata* Bl. *f. glauca* protocorms germinated by different fungi (Figure 2A and Table 1). Of these, JFGL-06 sprouted the highest number of protocorms, while JFGL-02 could not sprout. The length of the germinating protocorm obtained from the different *Mycena* fungi strains differed significantly (*p* < 0.05), from long to short, as control > JFGL-06 > JFGL-01 > JFGL-04 > JFGL-03 > JFGL-05 > JFGL-02. The weight of protocorms obtained from germinating seeds varied significantly (*p* < 0.05) among different strains. The descending order of protocorm weight was control, JFGL-06, JFGL-05, JFGL-01, JFGL-03, JFGL-04, and JFGL-02. The results showed that among all the *Mycena* strains, the length and weight of the germinated protocorms obtained from strain JFGL-06 were only smaller than the control, but the number of germinated protocorms from JFGL-06 was more than other strains. These results showed that JFGL-06 is more suitable for the cultivation of *G. elata* Bl. *f. glauca*. The JFGL-06 strain was tested for strain degeneration, and the same strain was inoculated for different generations, and the growth status of the strain was observed in order to determine whether the strain showed a decline in growth status and other conditions (Figure 2B). We will further explore whether JFGL-06 affects the absorption of elements in *G. elata* Bl. *f. glauca* protocorm.

**Table 1:** Comparison of germinating power of different germinating fungi.

Strain number	Number of protocorm (Number/100 cm^2^)	Average length of protocorm (cm)	Average weight of protocorm (mg)
CK	43.66 ± 3.21^e^	3.21 ± 0.06^g^	75.09 ± 1.86^f^
JFGL-01	26.00 ± 4.00^d^	1.81 ± 0.05^e^	24.44 ± 1.95^c^
JFGL-02	0.00 ± 0.00^a^	0.00 ± 0.00^a^	0.00 ± 0.00^a^
JFGL-03	16.67 ± 2.52^c^	1.65 ± 0.02^c^	23.42 ± 2.08^c^
JFGL-04	19.33 ± 3.06^c^	1.75 ± 0.03^d^	16.12 ± 1.06^b^
JFGL-05	11.33 ± 1.53^b^	1.56 ± 0.02^b^	37.04 ± 1.60^d^
JFGL-06	59.00 ± 4.00^f^	2.30 ± 0.01^f^	60.61 ± 3.87^e^

Data in the same column with different small letters mean significant difference (*P* < 0.05).

### 3.3. Analysis of absorption of nutrition elements in *G. elata* Bl. *f. glauca* co-planted with JFGL-06

Based on the excellent *Mycena* strains screened in the above study, inorganic elements were further examined by ICP-MS in primary bulbs and soil cultured by two *Mycena* fungi (control: commercial strain *Mycena*, treatment: strain JFGL-06). Two eight elements have a significant impact on plant growth and are present in high concentrations, namely B, C, N, Na, Mg, Al, Si, P, S, Cl, K, Ca, Cr, Mn, Fe, Co, Ni, Cu, Zn, Se, Be, Sc, As, Rb, Mo, Sn, I, and Ba (Table 2). The content of C, N, Na, Mg, Al, Si, P, S, K, Ca, and Fe in the samples of the two groups differed significantly. In the treated group, K was significantly higher in the *G. elata* Bl. *f. glauca* protocorms (184.85 mg/kg) than in the control group (90.67 mg/kg); the elemental contents of C, N, Na, Mg, Al, S, Cl, K, Ca, Fe were significantly different in the different soils of the two groups, The content of C, N, Na, Mg, S, Cl, K, Ca, Fe, in the treated *G. elata* Bl. *f. glauca* protocorms was significantly higher than that in the control group. The higher enrichment coefficients of the elements indicated that the plants were more absorbent of the elements, and there were significant differences in the enrichment coefficients of the elements in the two groups, including C, Na, Al, Si, P, Cl, Ca, and Cu. Only the enrichment coefficient of Ni was significantly higher in the treated *G. elata* Bl. *f. glauca* protocorms than in the control *G. elata* Bl. *f. glauca* protocorms (Table 2). The study revealed that JFGL-06 had an impact on the absorption of elements in *G. elata* Bl. *f. glauca* protocorms. Further research will be conducted to investigate the effect of JFGL-06 on microbial diversity in the soil surrounding these protocorms.

**Table 2:** Element contents (mg/kg) and enrichment factors in protocorm and soil under different treatments.

Elements	Control group protocorm	Control group soil	Enrichment factor of elements in control group	Treatment group protocorm	Treatment group soil	Enrichment factor of elements in treatment group
	(mg/kg)	(mg/kg)		(mg/kg)	(mg/kg)	
B	3.38 ± 0.58	1.28 ± 0.20	2.7 ± 0.73	2.33 ± 0.11	1.71 ± 0.63	1.54 ± 0.70
C	28793.30 ± 5983.56*	3630.96 ± 775.58	8.24 ± 2.81*	16154.97 ± 1610.91	5665.18 ± 2662.57*	3.39 ± 1.81
N	6308240.93 ± 36.72*	5382039.34 ± 355091.02	1.18 ± 0.12	5161894.63 ± 203054.44	5961823.10 ± 681713.96*	0.87 ± 0.09
Na	168.91 ± 397603.21*	67.87 ± 5.66	2.53 ± 0.76*	65.76 ± 15.18	83.37 ± 44.41*	0.87 ± 0.26
Mg	1360.06 ± 245.62*	1921.32 ± 198.53	0.71 ± 0.14	881.0 ± 160.79	1952.63 ± 619.98*	0.47 ± 0.11
Al	221.93 ± 36.55*	135.58 ± 177.40*	1.64 ± 1.08*	156.93 ± 84.20	146.78 ± 58.26	1.16 ± 0.62
Si	128.55 ± 40.37*	71.86 ± 2.73	1.78 ± 0.49*	82.72 ± 3.63	77.07 ± 4.56	1.07 ± 0.05
P	732.82 ± 158.46*	159.96 ± 19.91	4.69 ± 1.48*	483.25 ± 74.79	152.11 ± 49.56	3.4 ± 1.09
S	4649.90 ± 335.44*	3017.27 ± 142.11	1.54 ± 0.15	4170.25 ± 79.20	3317.63 ± 267.07*	1.26 ± 0.12
Cl	301.10 ± 85.19	91.31 ± 18.99	3.35 ± 1.02*	295.59 ± 62.79	117.19 ± 30.08*	2.58 ± 0.45
K	90.67 ± 3.00	1315.10 ± 74.92	0.07 ± 0.004	184.8 ± 86.93*	1741.86 ± 756.09*	0.11 ± 0.03
Ca	9574.28 ± 1929.86*	6412.80 ± 543.10	1.51 ± 0.38*	5057.96 ± 487.40	9497.73 ± 4135.15*	0.62 ± 0.31
Cr	1.16 ± 0.02	9.14 ± 0.47	0.13 ± 0.02	1.13 ± 0.59	9.93 ± 2.24	0.11 ± 0.04
Mn	19.34 ± 5.72	226.22 ± 14.92	0.08 ± 0.02	14.52 ± 7.16	223.43 ± 65.25	0.07 ± 0.02
Fe	504.37 ± 91.42*	9907.61 ± 361.31	0.05 ± 0.01	381.31 ± 157.14	11024.36 ± 3632.05*	0.03 ± 0.01
Co	0.40 ± 0.13	4.84 ± 0.21	0.08 ± 0.02	0.29 ± 0.16	5.16 ± 1.36	0.06 ± 0.02
Ni	0.67 ± 0.17	6.07 ± 4.07	0.14 ± 0.06	0.38 ± 0.42	0.66 ± 1.14	0.57 ± 0.02*
Cu	8.35 ± 3.20	4.48 ± 0.51	1.87 ± 0.73*	2.81 ± 0.43	5.70 ± 1.50	0.51 ± 0.09
Zn	11.89 ± 2.70	22.02 ± 1.95	0.55 ± 0.16	8.75 ± 1.69	26.14 ± 7.67	0.35 ± 0.10
Se	0.65 ± 0.04	0.75 ± 0.05	0.87 ± 0.11	0.54 ± 0.03	0.79 ± 0.11	0.68 ± 0.06
Be	0.01 ± 0.00	0.20 ± 0.01	0.05 ± 0.01	0.01 ± 0.00	0.22 ± 0.07	0.03 ± 0.002
Sc	0.16 ± 0.02	1.64 ± 0.08	0.11 ± 0.01	0.10 ± 0.03	1.63 ± 0.52	0.06 ± 0.01
As	0.11 ± 0.02	2.08 ± 0.19	0.05 ± 0.02	0.10 ± 0.04	2.52 ± 1.00	0.04 ± 0.01
Rb	6.47 ± 1.28	9.74 ± 0.46	0.67 ± 0.16	3.58 ± 0.40	8.89 ± 2.96	0.43 ± 0.13
Mo	0.11 ± 0.03	0.18 ± 0.01	0.61 ± 0.18	0.09 ± 0.03	0.25 ± 0.09	0.39 ± 0.13
Sn	0.02 ± 0.00	0.04 ± 0.01	0.53 ± 0.12	0.01 ± 0.01	0.05 ± 0.02	0.27 ± 0.05
I	0.27 ± 0.09	2.75 ± 0.24	0.09 ± 0.03	0.19 ± 0.05	3.04 ± 1.03	0.06 ± 0.01
Ba	15.36 ± 3.17	121.46 ± 4.09	0.13 ± 0.03	7.65 ± 0.54	119.17 ± 35.68	0.07 ± 0.02

*Indicates significant difference (*p* < 0.05) ± standard errors.

### 3.4. Composition and diversity analysis of soil microbial communities

**Figure 3:**
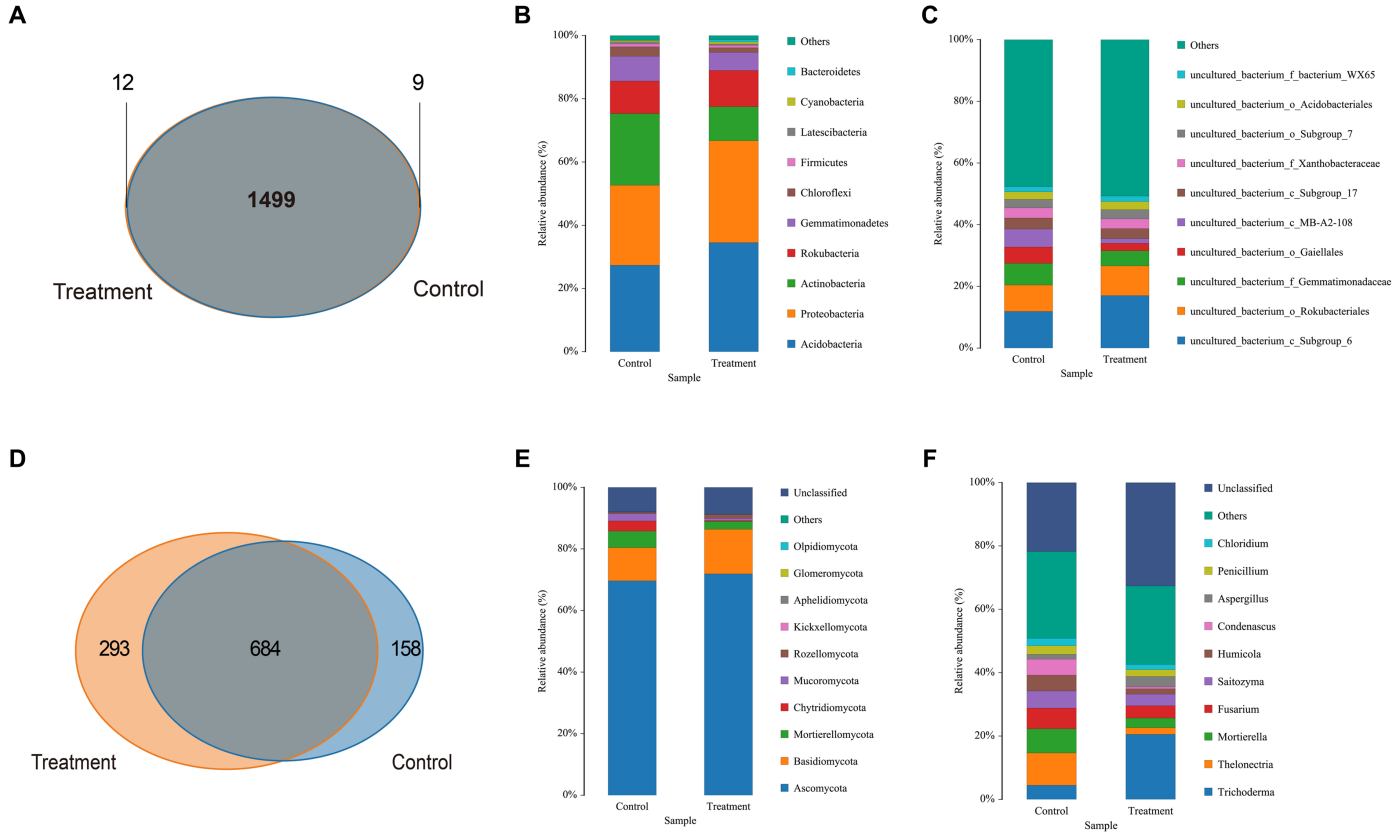
The composition of soil microbial community around *Gastrodia elata* Bl. *f. glauca* in two symbiotic its combination with commercial strains (control) and JFGL-06 (treatment). OTU count of bacteria **(A)** and fungi **(D)** in soil samples. Relative abundance of bacteria **(B)** and fungi **(E)** in each sample at the phylum level. Relative abundance of bacteria **(C)** and fungi **(F)** in each sample at the genus level.

The bacterial high-quality sequence coverage is over 94% and the fungal high-quality sequence coverage is over 98%. The dilution curve leveled off for each sample when the number of sequences was >4,000, indicating that the sample sequences were able to cover most of the species in the soil samples (Supplementary Figure S1). Venn diagrams were used to analyze the bacterial and fungal OTUs in both the control and treatment protocorms surrounding soil. The total number of bacterial OTUs was found to be 1,499, while the total number of fungal OTUs was 684. The number of fungi and bacteria that were specific to the treatment group was higher than that of the control group, as shown in Figures 3A,D. These indicates that strain JFGL-06 has increased the diversity of microorganisms in the surrounding soil of *G. elata* Bl. *f. glauca* protocorms.

Firstly, the relative abundance of soil bacteria and fungi surrounding *G. elata* Bl. *f. glauca* was observed at the phylum level under symbiosis with different *Mycena* strains. The dominant bacteria in the soil surrounding protocorms of *G. elata* Bl. *f. glauca* are mainly composed of *Acidobacteria*, *Proteobacteria*, *Actinobacteria*, *Rokubacteria*, *Gemmatimonadetes, Chloroflexi, Firmicutes, Latescibacteria, Cyanobacteria*, and *Bacteroidetes.* Compared to the control soil, the relative abundance of *Acidobacteria*, *Proteobacteria*, and *Rokubacteria* increased by 7.14, 6.92, and 1.17%, respectively, in the treatment soil; however, the phylum *Actinobacteria*, *Gemmatimonadetes*, and *Chloroflexi* decreased by 11.87, 2.11, and 1.58%, respectively (Figure 3B). The dominant fungi in the soil surrounding the protocorms of *G. elata* Bl. *f. glauca* consisted mainly of *Ascomycota, Basidiomycota, Mortierellomycota, Chytridiomycota*, *Mucoromycota, Rozellomycota, Kickxellomycota, Aphelidiomycota, Glomeromycota*, and *Olpidiomycota*. Compared to the control soil, the relative abundance of *Ascomycota* and *Basidiomycota* increased by 2.25 and 3.70%, respectively, and the *Mortierellomycota*, *Chytridiomycota*, *Mucoromycota* decreased by 2.80, 3.03, and 1.67%, respectively, in the treatment soil (Figure 3E).

Secondly, we found that at the genus level, the dominant bacteria in the soil surrounding the protocorms of *G. elata* Bl. *f. glauca* consisted mainly of uncultured-bacterium (Figure 3C). Compared to the control soil, the relative abundance of *uncultured_bacterium_c_Subgroup_6* and *uncultured_bacterium_o_Rokubacteriales* increased by 5.13 and 1.09%, respectively, in the soil surrounding protocorms of *G. elata* Bl. *f. glauca* in the treatment soil, *uncultured_bacterium_f_Gemmatimonadacea*, *uncultured_bacterium_o_Gaiellales* and *uncultured_bacterium_c_MB-A2-108* were reduced by 2.17, 2.84, and 4.32%, respectively. The dominant fungi at the genus level in the soil surrounding the protocorms of *G. elata* Bl. *f. glauca* were mainly composed of *Trichoderma, Thelonectria, Mortierella, Fusarium, Saitozyma, Humicola, Condenascus, Aspergillus, Penicillium*, and *Chloridium*. Compared to the control soil, the relative abundance of *Trichoderma* and *Aspergillus* increased by 16.08 and 1.76%, respectively, in the treatment soil, while the relative abundance of *Thelonectria, Mortierella, Fusarium, Saitozyma, Humicola*, and *Condenascus* decreased by 8.13, 4.57, 2.65, 1.71, 3.32, and 4.45% respectively (Figure 3F).

**Figure 4:**
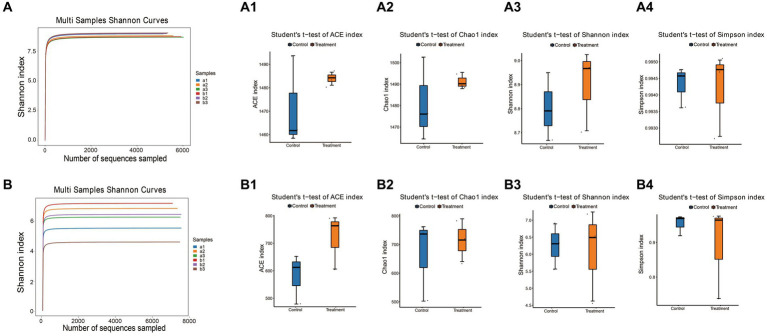
Analysis of soil microbial diversity around *Gastrodia elata* Bl. *f. glauca* in two symbiotic its combination with commercial strains (control, a) and JFGL-06 (treatment, b). The digital number represented three biological replicates for each sample. Shannon diversity index dilution curve of bacteria **(A)** and fungi **(B)** in soil samples. Histogram of differences between groups of soil bacterial diversity Ace index **(A1)**, Chao1 index **(A2)**, Shannon index **(A3)** and Simpson index **(A4)**. Histogram of differences between groups of soil fungal diversity Ace index **(B1)**, Chao1 index **(B2)**, Shannon index **(B3)**, and Simpson index **(B4)**.

**Figure 5:**
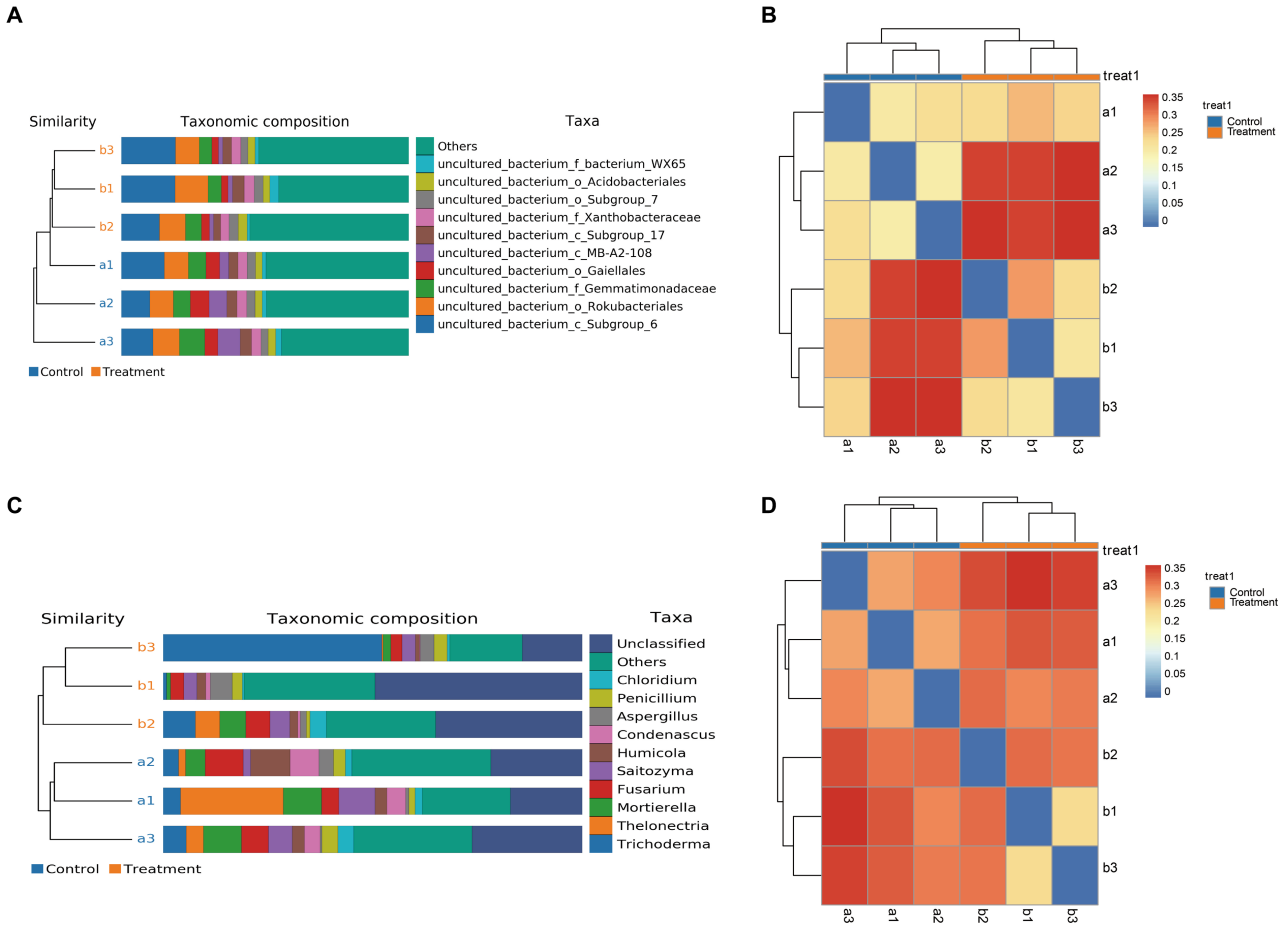
Analysis of soil microbial diversity around *Gastrodia elata* Bl*. f. glauca* in two symbiotic its combination with commercial strains (control, a) and JFGL-06 (treatment, b). The digital number represented three biological replicates for each sample. UPGMA Analysis of bacteria **(A)** and fungi **(C)** in soil samples. Cluster heatmaps of bacteria **(B)** and fungi **(D)** in soil sample.

Lastly, we measured the alpha diversity of microorganisms in *G. elata* Bl. *f. glauca* using multiple indices, including the Ace index, Chao1 index, Shannon index, and Simpson index (Supplementary Table S3 and Figures 4A,B). The Ace, Chao1 and Shannon indices increased in the treated group compared to the control in the soil bacterial samples (Figures 4A1–A4), and in the soil fungal samples, the Ace index was higher in the treated group than in the control (Figures 4B1–B4). The treatment soil increased the abundance and diversity of microorganisms in the soil compared to the control group, indicating that JFGL-06 strains could increase the abundance and diversity of soil microorganisms around the surrounding protocorms of *G. elata* Bl. *f. glauca*. We analyzed the beta diversity of soil microorganisms surrounding the protocorms of *G. elata* Bl. *f. glauca* using UPGMA clustering tree and heat map clustering analysis. (Figures 5A,C). At the genus level, the fungal communities in the controls and treatments formed distinct clusters with similar fungal abundance (Figures 5B,D). The results indicate that the JFGL-06 strain positively impacted the microbial abundance and diversity in the soil surrounding the protocorms of *G. elata* Bl. *f. glauca*. This could potentially contribute to an increase in yield for *G. elata* Bl. *f. glauca*.

### 3.5. The analysis of soil microbial differences

**Figure 6:**
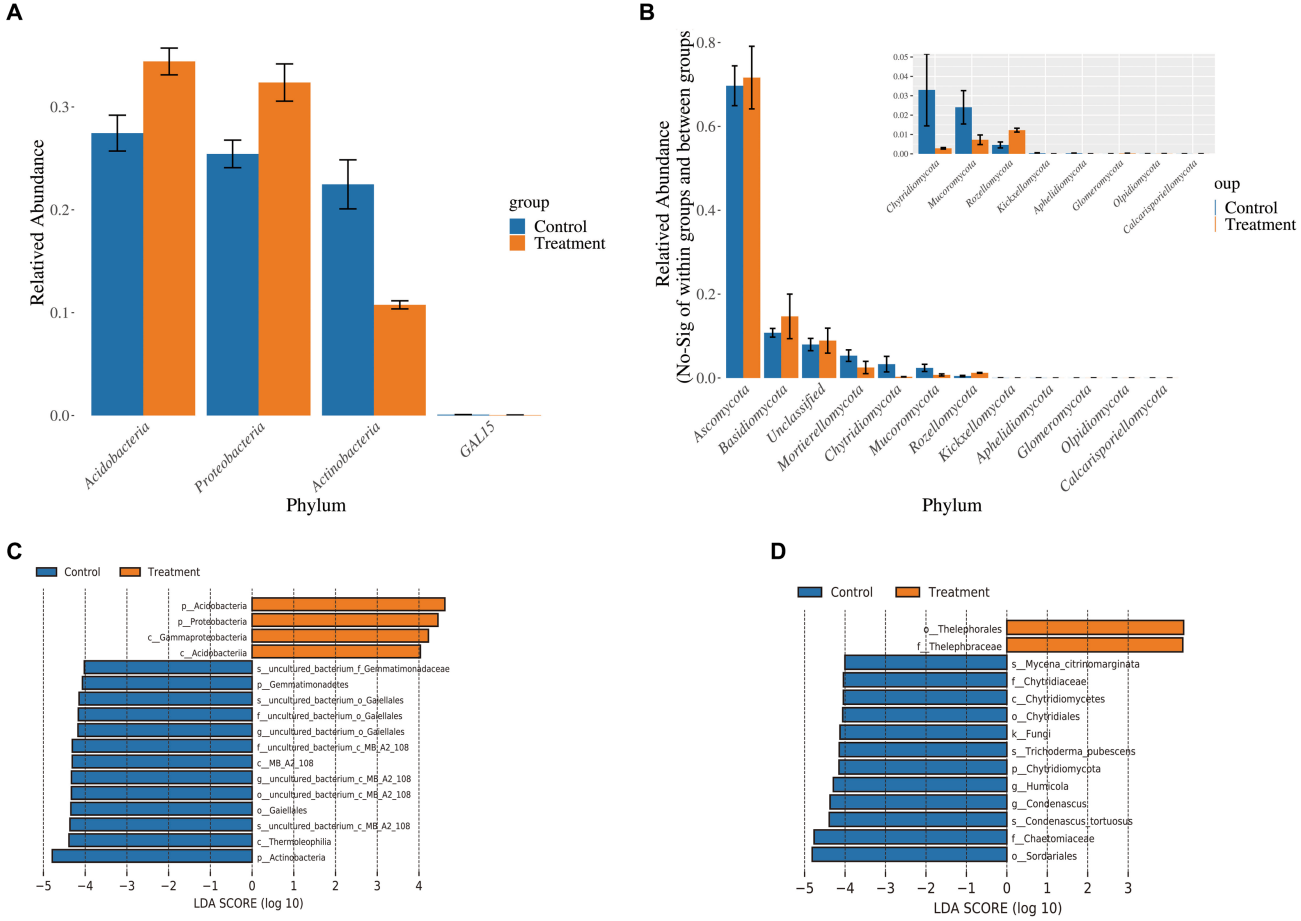
Difference analysis of soil microbiome around *Gastrodia elata* Bl. *f. glauca*. ANOVA analysis of bacteria **(A)** and fungi **(B)** in soil samples. Distribution histogram of LDA values of bacteria **(C)** and fungi **(D)** in soil samples.

Based on ANOVA analysis, we further explored the degree of significance of differences in the surrounding microbial communities of protocorms soil between the two groups of samples. Firstly, at the bacterial phylum level, *Acidobacteria, Actinobacteria*, and *Proteobacteria* were significantly different between groups (*p* < 0.05), with the relative abundance of *Acidobacteria* and *Proteobacteria* in the surrounding soil of the protocorms in the treatment group being higher (Figure 6A). At the fungal phylum level, *Ascomycota, Basidiomycota, Glomeromycota, Chytridiomycota*, and *Mucoromycota* were significantly different between treatment groups (*p* < 0.05), while the relative abundance of *Ascomycota* and *Basidiomycota* was higher in the surrounding soil of the protocorms in the treatment groups (Figure 6B). Secondly, we examined the contribution of differences in microbial abundance in different groups to the differences between groups by LEfSe analysis. Among the bacteria, *Actinobacteria* contributed the most difference in the control group and *Acidobacteria* contributed the most difference in the treatment group (Figure 6C). In contrast, among the fungi, *Sordariales* contributed the greatest difference in the control group and *Thelephorales* in the treatment group (Figure 6D). The results suggest that these four microorganisms are likely to be the key microorganisms causing variation in the yield of *G. elata* Bl., notably the *Thelephorales*, which also belong to the *Basidiomycota*. The JFGL-06 strain has been identified as a potential mechanism for enhancing both the yield and quality of *G. elata* Bl. *f. glauca*. This is achieved by improving the microbial community in the soil surrounding the protocorms of *G. elata* Bl. *f. glauca*.

### 3.6. PICRUSt2 functional prediction analysis of the soil

**Figure 7:**
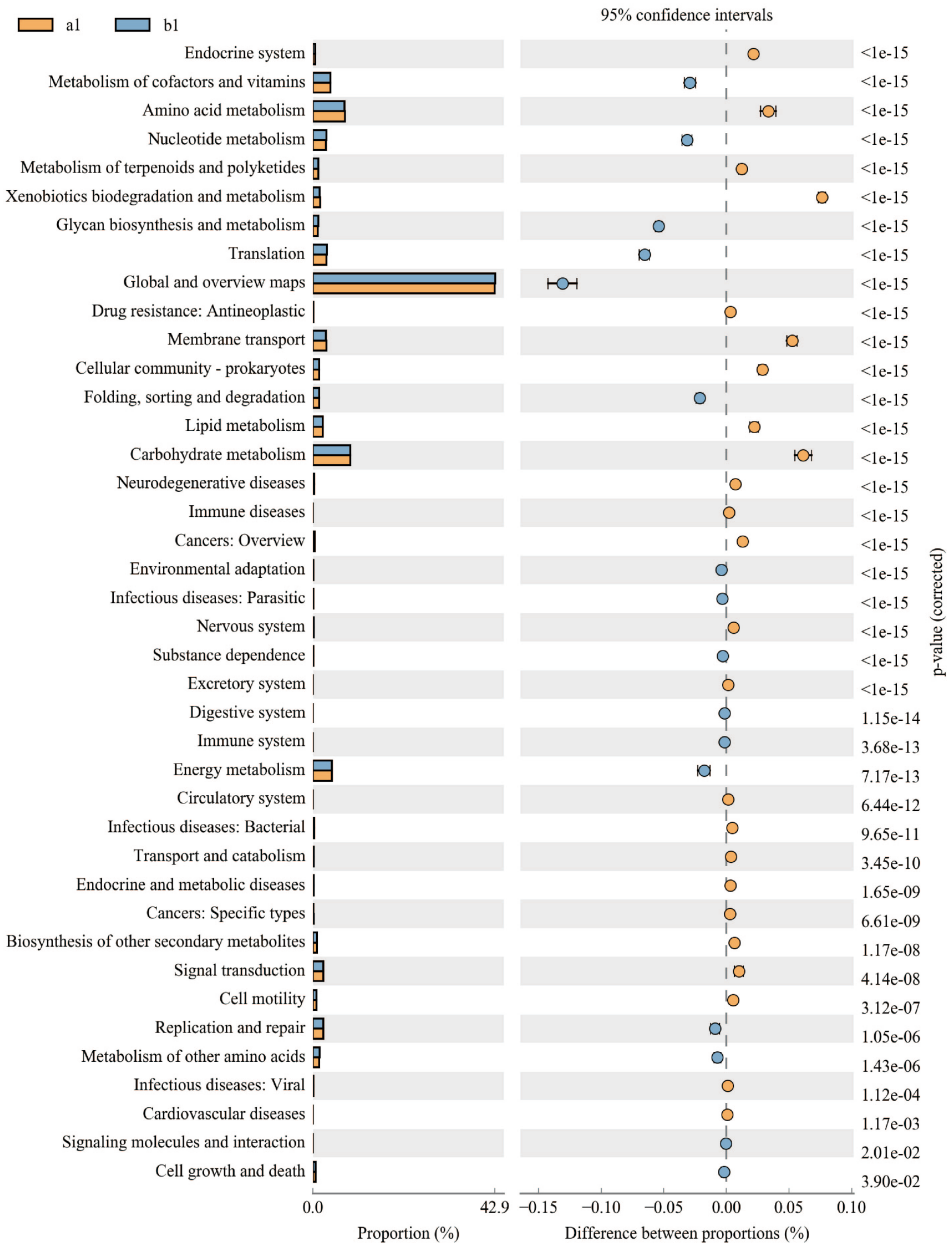
Difference analysis of KEGG metabolic pathways in soil bacteria around *Gastrodia elata* Bl. *f. glauca*.

PICRUSt2 is used to predict microbial community functions from marker gene sequencing. Upon further analysis of the microbiota’s functional changes, we discovered that out of the 40 secondary metabolic functions observed in the KEGG metabolic pathway at the second level, 16 were found to have significant differences between the control and treatment groups. Of these, nine secondary functions were predicted to have lower copy numbers in the treatment group than in the control group, including the Endocrine system, Amino acid metabolism, Metabolism of terpenoids, and polyketides, Xenobiotics biodegradation and metabolism, Drug resistance: Antineoplastic, Membrane transport, Cellular community-prokaryotes, Lipid metabolism, and Carbohydrate metabolism, but seven secondary function predicted genes had higher copy numbers in the concurrently treated group than in the control group, including Metabolism of cofactors and vitamins, Nucleotide metabolism, Glycan biosynthesis and metabolism, Translation, Folding, sorting and degradation, and Energy metabolism. The findings indicate that the JFGL-06 strain has an impact on the metabolic function of soil microorganisms present in the vicinity of *G. elata* Bl. *f. glauca*’s protocorms (Figure 7). This strain can serve as an alternative approach to enhance the yield and quality of *G. elata* Bl. *f. glauca*.

## 4. Discussions

*Gastrodia elata* Bl., a food and drug herb, is highly sought after in the current market due to its various effects, including promoting neural differentiation and delaying aging (Hsu et al., 2021; Yang et al., 2021), and its seed germination relies on *Mycena* fungi to provide nutrients and develop protocorms (Kim et al., 2006; Huang et al., 2018). *Mycena* fungi are widely distributed in China (Kim et al., 2006; Liu et al., 2015), and 39 species have been reported from the Changbai Mountain region of northeastern China alone (Cortes-Perez et al., 2019; Na and Bau, 2019a,b). *Mycena* is a diverse genus that includes a large number of species, but only a small number of species exhibit germination effects (Higaki et al., 2017; Na et al., 2022). Guo et al. isolated *M. dendrobii* from wild *Dendrobium officinale* and tested 12 orchid seeds with this strain for germination and found that the strain promoted germination of *G. elata* Bl. seeds. Liu et al. found that fungi from other genera (*Cephalosporium, Chaetomium, Ceratorhiza,* and *Epulorhiza*) promote the germination of *G. elata* Bl. seeds (Liu et al., 2010). Therefore, more *Mycena* fungi should be isolated and identified to enhance the production of *G. elata* Bl. However, there are many difficulties in isolating *Mycena* fungi. For example, *Mycena* fungi are small, but there are many methods to quickly screen for varieties that can promote the germination of *G. elata* Bl. seeds. At the same time, the fruiting body of the germination fungi exists for a short time and cannot correspond to the germination window period of *G. elata* Bl. seeds. The fungus *Mycena* was directly separated from the protocorm of *G. elata* Bl., and simultaneously, the technology of strain preservation was used so that a germination experiment could be carried out in the germination window period of *G. elata* Bl. seeds, which solves the problem of screening for the *Mycena* fungi capable of promoting the germination of *G. elata* Bl. seeds and lays the foundation for screening excellent strains. In this study, rDNA sequencing of the fungal ITS region was performed and a phylogenetic tree was constructed to distinguish between different isolates (Jacquemyn et al., 2011; Swift et al., 2019). Although the six strains isolated in this study belong to the same strain, they still enriched the germplasm resources of the symbiotic *Mycena* strains of *G. elata* Bl. *f. glauca.*

Excellent symbiotic strains play an important role in the normal growth and development of plants (Bidartondo and Read, 2008; Cely et al., 2016). *Mycena* fungi play a major role in the growth of *G. elata* Bl. and the different strains directly affect the yield and quality of *G. elata* Bl. Studies have shown that important indicators for screening good strains include the ability of *G. elata* Bl. seeds to obtain nutrients from *Mycena* fungi and germinate, after which the test continues, leading to a high rate of germination and stable germination momentum of *G. elata* Bl. seeds, which can be identified as a good strain (Mc et al., 2000; Zhang et al., 2012). The mycelial germination time, morphology, and growth and development rates of *Mycena* fungi vary, directly affecting their biomass, which in turn affects the germination rate of *G. elata* Bl. seeds, which is an important indicator for screening good *Mycena* fungi. This study adopted multiple indicators to evaluate the advantages and disadvantages of *Mycena* fungi, which can be more accurately screened for *Mycena* fungi and is of great value for the production of *G. elata* Bl. All six strains of *Mycena* fungi isolated in this study promoted the germination of *G. elata* Bl. seeds, whereas strain JFGL-06 had a faster growth rate and thicker mycelium, and the germination rate of *G. elata* Bl. seeds were better than that of the other strains, which was more suitable for the germination of *G. elata* Bl. *f. glauca* seeds.

In the mycorrhizal symbiotic relationship formed by most terrestrial plants and fungi on earth, symbiotic fungi can provide nutrients for the normal growth and development of plants (Tarnabi et al., 2020). Mycorrhizae can effectively improve the uptake of elements such as C, N, and P by plants, thereby promoting their growth and development (van der Heijden et al., 2015). For example, AMF can improve and enhance nutrient uptake in plants such as *Medicago truncatula* (Wipf et al., 2014), Leguminosae (Yang et al., 2016), and cereal crops (Barazetti et al., 2019). Various nutrients and minerals required by orchids during growth can be provided directly by mycorrhizal fungi (Yang et al., 2016). In this study, the JFGL-06 strain changed the contents of various elements in *G. elata* Bl. *f. glauca* protocorm and its surrounding soil. Compared with the commercial strain, it increased the content of C, N, Na, Mg, and other elements in the soil, as well as the content of K in the protocorm. Unfortunately, we did not specifically analyze each element. It is impossible to determine which specific pathways in plants are affected by the JFGL-06 strain; however, our study shows that the JFGL-06 strain improves the germination rate of *G. elata* Bl. *f. glauca* seeds by affecting the absorption and utilization of elements by *G. elata* Bl. *f. glauca.*

There is an interactive ecological relationship and nutrient cycle between *G. elata* Bl., *Mycena* fungi, soil, and soil microorganisms that maintain the balance between eukaryotes and soil, which, when disturbed, can affect plant growth (Duran et al., 2018; Shao et al., 2018; Swift et al., 2019). By analyzing the ecological environment of *G. elata* Bl. production area, the quality of the *G. elata* Bl. was mainly influenced by the soil microbial community, organic matter content, and pH of *G. elata* Bl. growth locations (Waud et al., 2016). Studies have shown that *Lactobacillus* can promote organic matter decomposition and facilitate plant-microbe interactions (Xia et al., 2011). *Bradyrhizobia* can also promote plant growth by improving the plant inter-root environment (Meena et al., 2018) and *Pseudomonas* are biocontrol microorganisms and dominant bacteria for healthy *G. elata* Bl., with strong control effects against root rot and wilt, etc. (Malandraki et al., 2008; Naik et al., 2008; Tran et al., 2008). The soil microbial community structure differed considerably between the wild and cultivated *G. elata* Bl. growing sites and the characteristics and traits of the soil microbial composition and physicochemical properties of *G. elata* Bl. cultivated with different fungal species. However, there is little scientific certainty as to whether different symbiotic strains may affect the community structure and function of surrounding soil microorganisms. In this study, the relative abundances of *Acidobacteria*, *Proteobacteria*, and *Trichoderma* increased in the soil surrounding *G. elata* Bl. *f. glauca* co-planted with JFGL-06, while the relative abundances of *Actinobacteria, Mortierella*, and *Fusarium* decreased compared to the commercial strain. Interestingly, *Acidobacteria* possesses genes associated with various metabolic pathways that regulate biogeochemical cycles, break down biopolymers, secrete extracellular polysaccharides, and promote plant growth. *Proteobacteria* are positively correlated with ammonium nitrogen levels in the soil, and *Trichoderma* is considered an important biocontrol fungus that can be used to prevent plant diseases caused by fungi, such as *Humicola* and *Fusarium. Actinobacteria*, *Mortierella*, and *Fusarium* are often associated with plant diseases. This result is consistent with the above-mentioned role of mycorrhizal microorganisms in antagonizing pathogenic microorganisms and enhancing beneficial microorganisms in the soil. Subsequently, JFGL-06 was tested with different symbiotic and antagonistic microorganisms to further determine the role of JFGL-06, which was also a limitation of this study. However, this study provides a new way to explore the impact of symbiotic strains on the diversity of plant rhizosphere microorganisms.

Notably, changes in metabolic pathways through functional genes in microbial communities in response to environmental changes, such as changes in abundance owing to the deletion of functional genes, can subtly affect the growth and development of host plants (Chen et al., 2020). In this study, the relative abundance of metabolic pathway functional genes, such as metabolism of cofactors and vitamins, nucleotide metabolism, glycan biosynthesis and metabolism, translation, and energy metabolism increased, and the relative abundance of metabolic pathway functional genes, such as amino acid metabolism and xenobiotic biodegradation and metabolism decreased. Metabolic pathways, such as nucleotide metabolism, amino acid metabolism, and metabolism of cofactors and vitamins are commonly associated with plant growth, and xenobiotic biodegradation and metabolism, translation, and energy metabolism pathways are even more relevant to plant apoptosis and disease resistance (Palmieri et al., 2022). These results indicated that the JFGL-06 strain improved the microbial metabolic function of the soil surrounding *G. elata* Bl. protocorms, which is different from the commercial strain. This is also one of the mechanisms by which it improves nutrient absorption and disease resistance in *G. elata* Bl., thus enhancing *G. elata* Bl yield and providing a reference basis for the subsequent optimization of high-yielding *G. elata* Bl. systems.

## 5. Conclusion

In this study, six isolates were obtained all of which were identified as *M. purpureofusca.* The JFGL-06 strain proved to be the optimum *Mycena* fungus for increased the nutrient content of *G. elata* Bl. *f. glauca* protocorms and the soil around them, especially the K content of the bulbs and C content of the soil. It also improved the diversity of the soil microbial community, particularly by enriching the diversity of soil fungi in the *Basidiomycota*. Finally, the metabolic functions of soil microorganisms were improved, especially important metabolic pathways such as energy metabolism and nucleotide metabolism. Therefore, JFGL-06 is an excellent strain for *G. elata* Bl. *f. glauca*, which provides new ideas for research to improve the yield and quality of *G. elata* Bl.

## Data availability statement

The datasets presented in this study can be found in online repositories. The names of the repository/repositories and accession number(s) can be found in the article/Supplementary material.

## Ethics statement

The *Mycena* isolated and identified in this study was provided by our laboratory, and the author has the right to use it without ethical and legal issues. The commercial strain *Mycena* used was purchased from Jingzhen Gastrodia Development Co., Ltd. (Baishan City, Jilin Province, China). The laboratory experiments were conducted in accordance with relevant l legislation and under permission. All methods were performed in accordance with relevant guidelines and regulations in this study.

## Author contributions

YG contributed to research conception, material collection, and writing guidance. PZ, YZ, and ZH provided technical support. YL assisted in experiment conduction. EY conducted the experiments and manuscript writing. All authors read and approved the final manuscript.

## Footnotes

^1^ http://www.arb-silva.de

^2^ https://unite.ut.ee/

## Supplementary material

### Supplementary Figure S1

Sequence analysis of soil microorganisms around *Gastrodia elata* Bl. *f. glauca* in two symbiotic its combination with commercial strains (a) and JFGL-06 (b). The digital number represented three biological replicates for each sample. Bacteria **(A)** and fungi **(B)** dilution curves of soil samples.
