# Supplementary figures and images for: An exploration of mechanism of high quality and yield of Gastrodia elata Bl. f. glauca by the isolation, identification, and evaluation of Mycena
Source: Front Microbiol. 2023 Oct 19;14:1220670. doi: 10.3389/fmicb.2023.1220670 (PMC10620705; doi:10.3389/fmicb.2023.1220670)

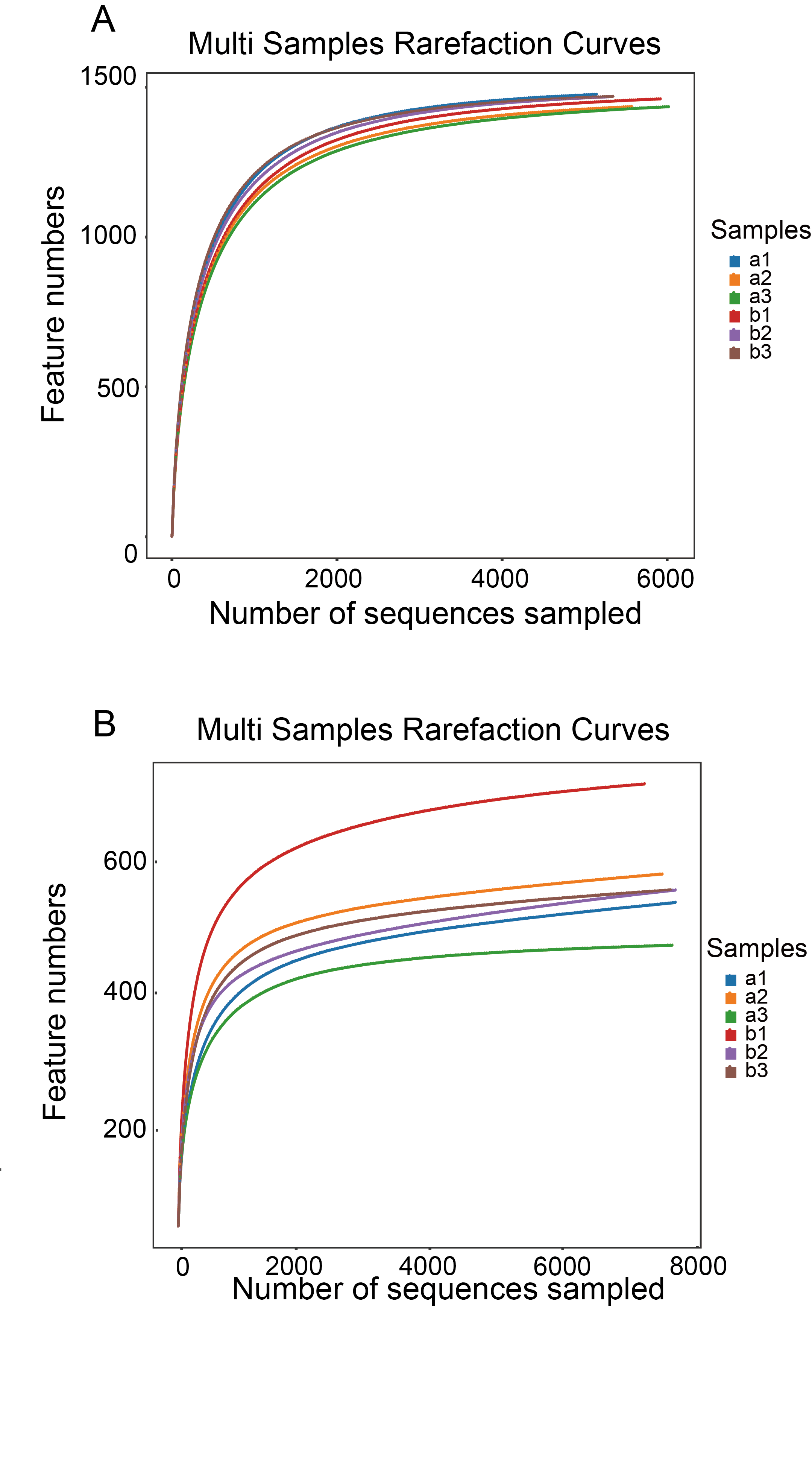

Supplement: Supplementary file 4 [file Image_1.png]
